# Wearable cardioverter-defibrillator as bridging to ICD in pediatric hypertrophic cardiomyopathy with myocardial bridging – a case report

**DOI:** 10.1186/s12887-020-02113-w

**Published:** 2020-05-11

**Authors:** Stefan Kurath-Koller, Hannes Sallmon, Daniel Scherr, Egbert Bisping, Ante Burmas, Igor Knez, Martin Koestenberger

**Affiliations:** 1 Division of Pediatric Cardiology, Department of Pediatrics, Medical University Graz, Graz, Austria; 2 Department of Pediatric Cardiology, Charité – Universitätsmedizin Berlin, corporate member of Freie Universität Berlin, Humboldt-Universität zu Berlin, and Berlin Institute of Health, Berlin, Germany; 3 Division of Cardiology, Department of Internal Medicine, Medical University Graz, Graz, Austria; 4 Division of Cardiac Surgery, Department of Surgery, Medical University Graz, Graz, Austria

**Keywords:** Hypertrophic cardiomyopathy, LifeVest, Ventricular tachycardia, Myocardial bridging

## Abstract

**Background:**

There is only limited experience with wearable cardioverter-defibrillators (WCD) in pediatric patients. We report on the successful application of a WCD in an adolescent patient with hypertrophic cardiomyopathy and myocardial bridging.

**Case presentation:**

A 15-year-old girl presented with a history of recurrent syncope, dyspnea, and vertigo with exercise. Diagnostic work-up revealed non-obstructive hypertrophic cardiomyopathy and signs of myocardial ischemia with exercise. Given this high-risk constellation, the patient was scheduled for prophylactic implantation of an implantable cardioverter-defibrillator (ICD). One month after initial presentation and days prior to the planned ICD implantation, the patient collapsed during an episode of sustained ventricular tachycardia (VT) while running. VT was terminated by WCD shock delivery. Following this event, computerized tomography scan revealed myocardial bridging of the left anterior descending coronary artery causing a 90% stenosis in systole. After coronary surgery, life threatening arrhythmias have not recurred, but due to progressive heart failure, the patient underwent successful heart transplantation after 2 years.

**Conclusions:**

The reported case highlights the importance and applicability of WCDs and the potentially malign nature of myocardial bridging in pediatric high-risk patients.

## Table of contents summary

An adolescent girl suffering from HCM and myocardial bridging of the LAD causing a 90% stenosis during systole was effectively defibrillated by a wearable cardioverter-defibrillator.

## Background

Implantable cardioverter-defibrillators (ICDs) represent a well established treatment option for patients at high risk for sudden cardiac death (SCD). Data supporting the efficacy in preventing SCD by ICDs have been shown by several randomized-controlled studies [1–4]. However, there are clinical scenarios where the use of an ICD is temporarily not feasible or implantation criteria are not yet met. The wearable cardioverter-defibrillator (WCD) is a temporary non-invasive device used for prevention of SCD in presumed high-risk patients suffering from potentially reversible conditions and may be used when implantation criteria for an ICD are not met [5]. Data on the use of WCDs as bridging to ICD implantation in high-risk pediatric patients with hypertrophic cardiomyopathy are limited. We report on the use of a WCD in a 15-year-old girl suffering from ventricular tachycardia (VT) due to myocardial bridging and non-obstructive hypertrophic cardiomyopathy.

## Case presentation

**Fig. 1:**
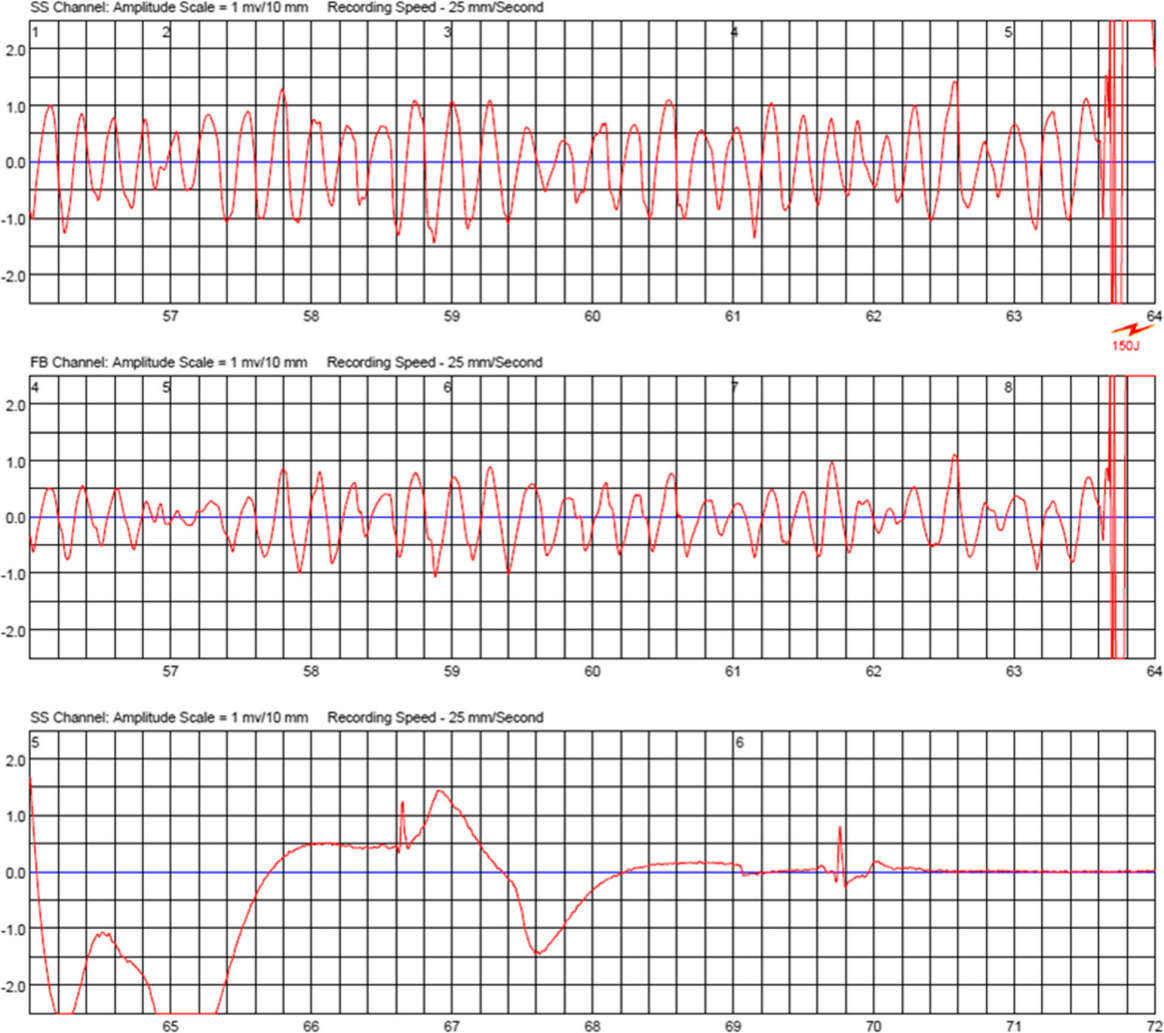
Sudden onset polymorphic ventricular tachycardia. After 30 s the LifeVest delivered an appropriate shock, converting ventricular tachycardia into sinus rhythm

**Fig. 2:**
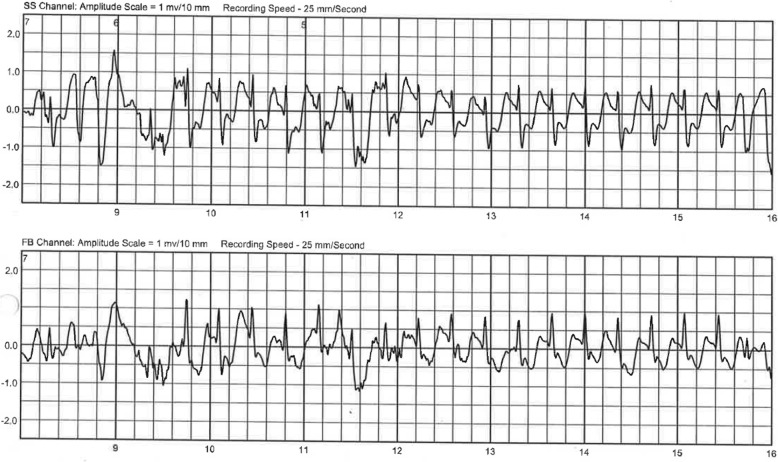
Sinus tachycardia (160 bpm) with ST-segment depression consistent with myocardial ischemia, which was then followed by sudden onset polymorphic ventricular tachycardia

**Fig. 3:**
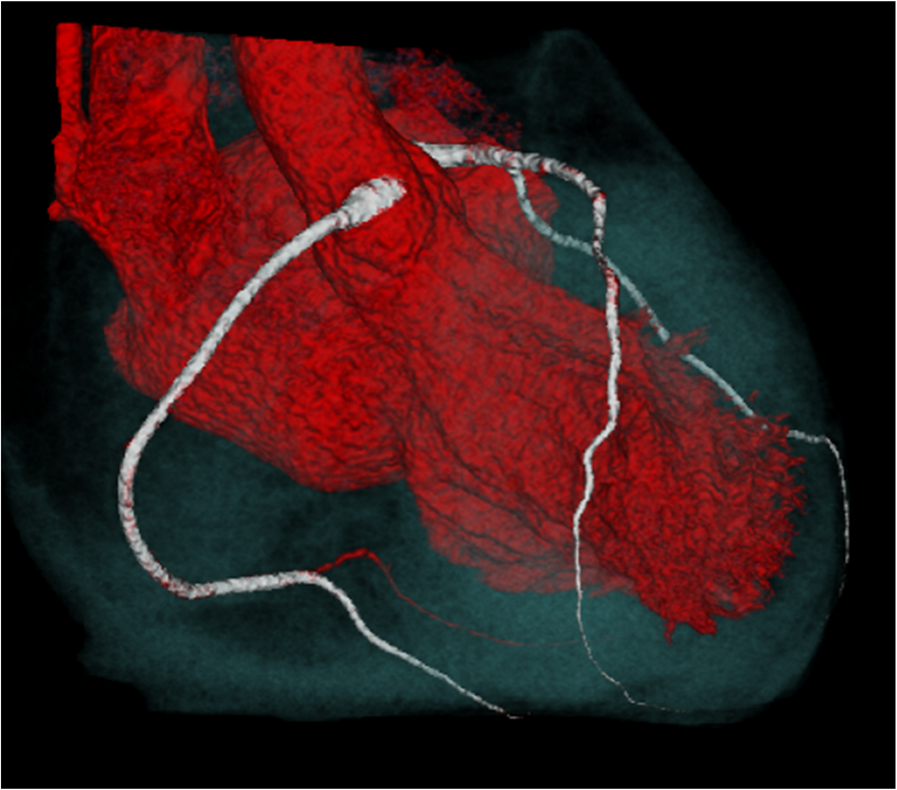
Three dimensional reconstruction of the computerized tomography scan and angiography depicting myocardial bridging of the left anterior descending coronary artery

A 15-year-old girl presented a history of recurrent syncope, dyspnea and dizziness. Echocardiography revealed a non-obstructive hypertrophic cardiomyopathy with a septal thickness of 25 mm (z-score + 17) and impaired diastolic function. Repeated Holter ECG monitoring revealed no life threatening arrhythmias. The patient was started on beta-blockers (metoprolol). Risk stratification was performed according to the 2014 ESC guidelines [6] estimating a 5-year risk for sudden cardiac death (SCD) of 7%, indicating that an ICD should be considered. Throughout the process of finalizing diagnostic work-up and decision making, we instructed the girl for meticulous use of a wearable cardioverter-defibrillator (LifeVest, ZOLL, Pittsburgh, Pennsylvania, USA). Further work-up included genetic testing, revealing a TNNC-1 gene mutation known to be related to hypertrophic cardiomyopathy [7]. However, family history was unremarkable in regard to sudden cardiac death or cardiomyopathy. Exercise testing (bicycle ergometer) resulted in angina symptoms and ST-segment depression in the left precordial leads, leading to a maximum performance of 1.2 W/kg. Symptoms resolved quickly after discontinuing exercise. Magnetic resonance imaging (MRI) and late gadolinium enhancement revealed areas of lower perfusion and fibrosis. The patient was scheduled for implantation of an ICD system given her high-risk constellation. Five days prior to the scheduled ICD implantation, after wearing the WCD for 6 weeks, the patient was admitted after experiencing another syncope with successful termination of a sustained VT by the WCD. During that episode she was running a very short distance to catch the school bus but then collapsed inside the crowded vehicle. After shock delivery by the WCD she recovered very quickly and was admitted to the hospital completely uncompromised. The ECG documented by LifeVest revealed severe ischemic changes right before initiation of ventricular tachycardia. Figure 1 shows VT and shock delivery by the WCD on the ECG tracing obtained by LifeVest. The ischemic changes (Fig. 2) prompted a computerized tomography (CT) scan of the coronary arteries. Subsequent coronary angiography revealed myocardial bridging of the left anterior descending coronary artery (LAD) causing a 90% stenosis in systole (Fig. 3 shows a 3D reconstruction from the CT scan). The patient underwent surgical unroofing of the LAD and a left internal mammarian bypass proximal to the bridged LAD was performed. Due to sustained VT, low cardiac output and low perfusion of the distal portion of LAD, a venous bypass distally to the bridged artery was performed during the same session. Postoperatively she needed 3 days of mechanical cardiac support and recovered slowly. Finally, an ICD was implanted. Following the single event of VT, life threatening arrhythmias have not recurred. Due to progressive restrictive heart failure and increasing severe angina symptoms following occlusion of the left mammarian artery bypass, she successfully underwent heart transplantation 2 years after her first event.

## Discussion and conclusions

The American Heart Association released guidelines on the indications of WCDs in 2016 [8]. WCD use was approved by the FDA in 2015 for children with a minimum weight of 42 pounds and a minimum chest circumference of 26 in., resembling an 8 year-old. Currently, LifeVest (ZOLL, Pittsburgh, Pennsylvania, USA) is the only WCD approved for clinical use. It has built-in electrodes for rhythm sensing, and pads to deliver a shock if necessary [9, 10]. In certain situations, when criteria for an implantable ICD are not entirely met and/or a transient risk for SCD exists, the WCD system represents a non-invasive but potentially life-saving treatment option. This is of particular interest in the pediatric age group, where criteria for ICD implantation are often not fulfilled and/or improvements of the condition is expected. Data on the use of WCDs in pediatric patients still remain scarce and additional evidence on the use of WCDs in pediatric patients is warranted. In addition, risk stratification for SCD was established in the adult population [11], while risk stratification for SCD in the younger pediatric population is less well validated. Thus, WCD indications might be applied less rigidly in children below 16 years of age when compared to adults. By applying the above mentioned adult risk stratification [11] to our adolescent patient, we found our patient to have an indication for an ICD and chose the WCD system as a protective bridge to ICD implantation.

Acceptance of treatment, by patients and parents, is an important factor and is probably higher for WCDs than for ICDs or subcutaneous cardioverter defibrillators (SICDs). Furthermore, ICDs show relatively high rates of complications in pediatric patients, most commonly related to lead problems which potentially have significant impact on the patient’s quality of life [12, 13]. Acceptance of treatment and comfort when waring the WCD system seems of outmost importance considering that the longest possible wearing time per day must be achieved in order to obtain maximum efficiency. WCD systems seem to be highly effective in terminating VT/VF in pediatric patients and inappropriate shock deliveries are infrequently observed [5].

Myocardial bridging is considered a benign variation of coronary development present in about 25% of the population. However, it may cause severe cardiac conditions, especially with underlying heart disease [14]. In HCM, signs of myocardial ischemia should trigger evaluation for myocardial bridging as a potentially treatable cause, and due to the risk of SCD, WCD use should be considered as a transient treatment option until surgical relief is performed. In our case, ischemic findings on the WCD tracing prior to shock delivery helped establish the diagnosis of myocardial bridging.

In conclusion, this case reports on the use of WCD as a bridge to ICD in a high-risk pediatric patient with HCM and myocardial bridging. The WCD allowed for primary protection from SCD while awaiting further diagnostic work-up.

## Data Availability

Not applicable.

## Abbreviations

ICD: Implantable cardioverter defibrillator
HCM: Hypertrophic cardiomyopathy
FDA: Food and drug administration
LAD: Left anterior descending coronary artery
SCD: Sudden cardiac death
SICD: Subcutaneous implantable cardioverter defibrillator
WCD: Wearable cardioverter defibrillator
